# Leaking of 316L stainless steel heat exchanger tubes in carbon dioxide process

**DOI:** 10.1038/s41598-025-21376-w

**Published:** 2025-10-07

**Authors:** Manar Mansour, Waleed Khalifa

**Affiliations:** Department of Metallurgical Engineering, Faculty of Engineering, Cairo University, Giza, Egypt

**Keywords:** Corrosion, Stainless steel 316L, Heat exchanger, Tube leaking, Dew point, Flue gas, Partial pressure, H_2_SO_4_, HCl, HF, H_2_CO_3_, Engineering, Materials science

## Abstract

This study covered a premature leaking of stainless steel 316L heat exchanger tubes. Leaking occurred in a cooling process of carbon dioxide gas mixture from 140 to 55°C. Several leaks were observed after short service life of 70 days. A systematic investigation of the rapid degradation was achieved through a multi-angle approach backed by comprehensive analytical techniques: including visual inspection, optical microscopy, SEM/EDS, and chemical analysis. In addition, valuable insights were obtained from the dew point modeling of condensed acids. The key finding and novelty of this study is the identification of the synergistic dew point corrosion by HCl and HF, and its role in tube damage. Recommendations for higher performance alloys were addressed as well. The insights and understanding presented in this work are believed to be of significant value for both academic and industrial readers.

**Supplementary Information:**

The online version contains supplementary material available at 10.1038/s41598-025-21376-w.

## Introduction

Austenitic stainless steels are used in wide engineering and industrial applications due to their desired properties^1^. High temperature resistance, high strength, excellent toughness and good electrical resistivity are some of the desirable physical properties of the austenitic stainless steels, besides their high corrosion resistance^2,3^. The ability to form the passive layer provides the austenitic stainless steels with exceptional corrosion resistance. However, corrosion is still the uttermost challenge facing these alloys when exposed to sever corrosive environments^4^. The 316 stainless steel is a chromium-nickel-molybdenum alloy, that is developed by adding molybdenum to enhance the corrosion and pitting resistance, particularly in chloride rich environments^5,6^. The 316L is a version of the 316 austenitic stainless steel alloy, characterized by extra low carbon content, as denoted by the letter L^7^, and was developed mainly for welding applications.

The 316L is used in heat exchangers, underground pipelines and in coastal areas due to its good combination of mechanical properties, good formability and corrosion resistance^8,9^. It was found that the 316 stainless steel grade was more corrosion resistant than the austenitic stainless steel grade 304L. The 316 alloys can be used in different corrosive media, with limited chloride levels, and in mild to moderately oxidizing and reducing environments^10^. The low carbon content in 316L decreases the susceptibility to sensitization^11^. It was also found that when the passive layer of austenitic stainless steel is broken at local spots, the chlorides of the corrosive media would accelerate the pitting rate^12^.

Low temperature corrosion or dew point corrosion is one of the most detrimental forms of corrosion facing the heat exchangers, preheaters and exhaust transfer systems, while being in service^13–15^. This phenomenon occurred due to the condensation of acidic compounds on the surface of the heat exchanger tubes. These compounds cause serious corrosion damage to metal surface and reduce the efficiency of the heat exchanger^16^. The compounds usually result from the combustion process of fuels enriched with sulfur and other impurities such as chlorine. The transformation into acidic vapors such as H_2_SO_4_, and HCl occurs via reaction with the water vapor. The condensation of acidic vapors on the steel surface takes place during the decrease of flue gas temperature^17,18^.

In the current study, two tube-type heat exchangers suffered localized degradation, leaking and malfunctioning, shortly after process commissioning. Both units were used for cooling a process gas, which is a mixture of the CO_2_ resulting from a lime calcination furnace, and the flue gas of combustion. The two units exhibited leaking of the cooling tubes. The leaking tubes were made of the 316L stainless steel. The leaking occurred rapidly during a service period of about 70 days, and, thus, deserved an investigation.

The study systematically addresses the real-world problem of rapid degradation of tube materials in the heat exchanging environment. This was achieved through a multi-angle approach supported by comprehensive analytical techniques, including: visual inspection, optical microscopy, SEM/EDS, chemical analysis, and dew point modeling. Apart from the previous works, which mainly focused on the dew point corrosion by sulfuric acid condensation, the current work identified the effects of synergistic dew point corrosion caused by the HCl and HF condensation. It is believed that these effects are insightful and novel. In addition, the insights and understanding presented in this work, through the combination of a real-world case investigation with predictive modeling, are believed to be of significant value for both academic and industrial readers.

## Service history and background data

Two heat exchangers were used in a process industry for cooling the CO_2_ gas resulting from lime calcination process. The two units exhibited malfunctioning and leaking, at the tube surfaces. The external surfaces of the tubes were in contact with the CO_2_ gas, while the internal surfaces were in contact with the cooling water. While being in service for only 70 days, the leaks occurred, therefore, it is considered a premature failure. Both units were put out of service to investigate the root cause of the problem. The service conditions of these units are given in Table 1.

**Table 1:** Service conditions of the heat exchangers.

Pressure of cooling water	Pressure of CO_2_	Level of CO_2_, %	Level of CaO or CaCO_3_	Temperature, °C			
				Inlet water	Exit water	Inlet gas	Exit gas
Max. 4 bar	250 mbar	18% CO_2_, 82% air	10 mg/m^3^ of CO_2_	25°C	47°C	120–140°C	55°C

The name plate information of the heat exchangers is shown in Table 2. The heat exchangers were designed and manufactured according to ASME boiler and pressure vessel code, section VIII, division 1^19^. The date of hydro test was May 2021, while the first operation was in July 2021. The heat exchangers were drained from water directly after the hydro test. The hydro test water and cooling water were condensate water.

**Table 2:** Name plate information of the heat exchangers.

Component	Pressure, bar		Temperature, °C		Volume, liter	Stress relieve
	Design	Test	Design	Test		
Tubes	0/7	13	0/185	AMB	13,100	No
Shell	0/0.5	5.5	0/185	AMB	14,450	No

## Materials and methods

**Fig. 1:**
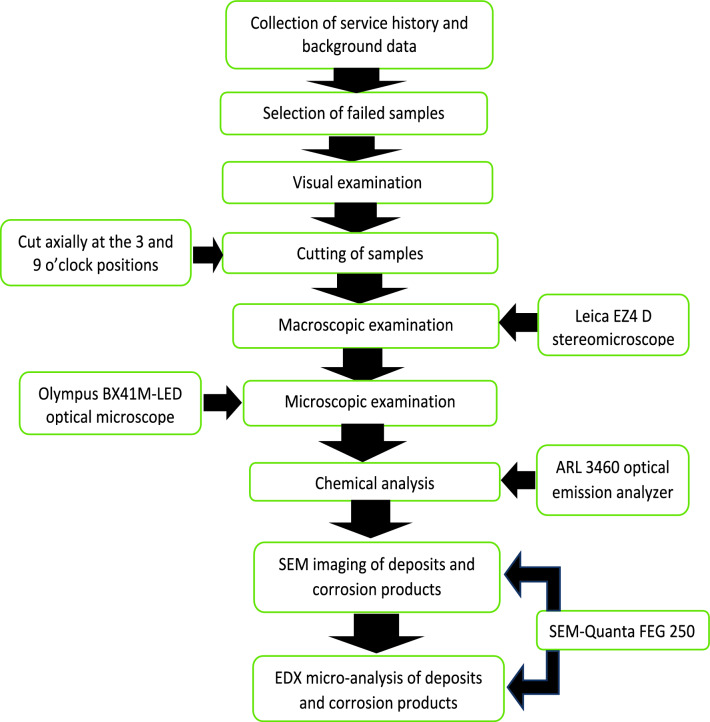
Flowchart illustrating the main tests and examination steps used in the current study.

Figure 1 shows a flowchart of the main examination steps of the current study. The material of the tubes is the stainless steel grade 316L. The study started with the collection of background data and service history, then the selection of samples. Visual examination was performed primarily with naked eye. The tubes were carefully examined for manufacturer markings, abuse, heat effects, corrosion scaling and cracking^20^. The overall equipment was visually inspected before cutting the samples for further examination. Photos were taken for the equipment and the samples as recommended in the literature^21^.

Macroscopic examination was done using Leica EZ4 D stereomicroscope. The whole external surfaces of the tubes were examined. Then, the samples were cut axially into two halves at the 3 and 9 o’clock positions. Both upper and lower internal and external surfaces were examined, as well. Metallographic specimens were prepared using silicon carbide grinding papers and polishing with alumina paste. Olympus BX41M-LED optical microscope was used for the microscopic examination, in the as-polished condition, and after etching. The chemical composition of the samples was determined using the ARL 3460 optical emission analyzer, and then compared with the relevant specifications. The scanning electron microscope (SEM—the Quanta FEG-250 model), and the energy dispersive spectroscopy (EDS) were used to image and analyze the surface deposits and corrosion products. The SEM examination was done using an accelerating voltage of 30,000 kV, and magnification ranges from 120 to 4000 X. Images and EDS analysis were taken via the secondary electron examination mode using the LFD and the ETD detectors. Several locations of corrosion damage were imaged and analyzed on the surface of leaking tubes.

## Results

The results of the study are shown in the following sections of the article.

### Inspection visit

**Fig. 2:**
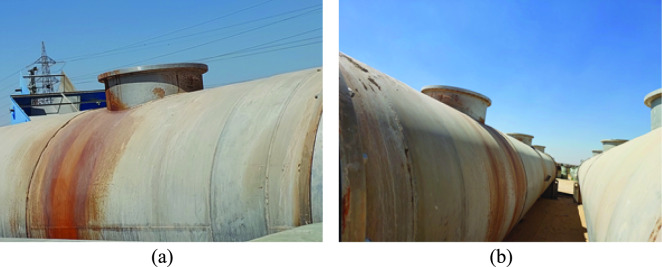
Images of the heat exchangers: (**a**) gas entrance, and (**b**) side walls.

**Fig. 3:**
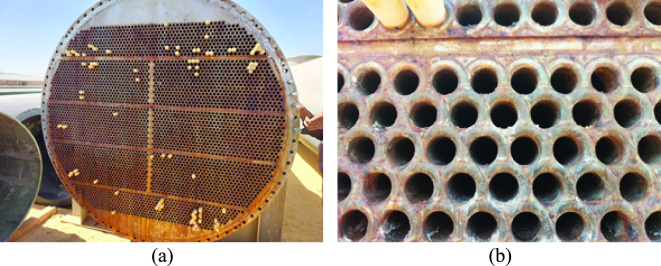
Images of the tube sheet of the leaking heat exchangers: (**a**) general view showing plugging of the leaking tubes, and (**b**) close-up view showing the welding of tubes to tube sheet.

During the inspection visit, the overall conditions of the leaking heat exchangers were assessed. In addition, samples for laboratory examination were selected. The main points of achievement in the inspection visit are collecting background data and technical information. Part of this information was presented earlier in the “Service history and background data” section of this article. Additional photographic images are shown in Figs. 2 and 3.

Figure 2 shows the two heat exchangers, which were put out of service, and intended for failure analysis investigation. Figure 3 shows the tube sheet of one of the heat exchangers. Figure 3 (a) shows that several tubes were plugged because of leaking, while Fig. 3 (b) shows the good appearance of tube welds at the tube sheet. According to the manufacturer data, each heat exchanger contains 3800 stainless steel tubes. It was reported that leaking occurred at the free surface of tubes, away from tube welds at tube sheet.

### Visual examination of the selected samples

The visual examination is one of the most useful tools of damage and failure investigations. It enables the researcher to determine whether the damaged component has been exposed to corrosion, wear or mechanical abuse; or damaged because of manufacturing defects. The visual examination is achieved by the unaided eye using certain tools such as cameras, magnifying glasses, camcorders and other tools.

**Fig. 4:**
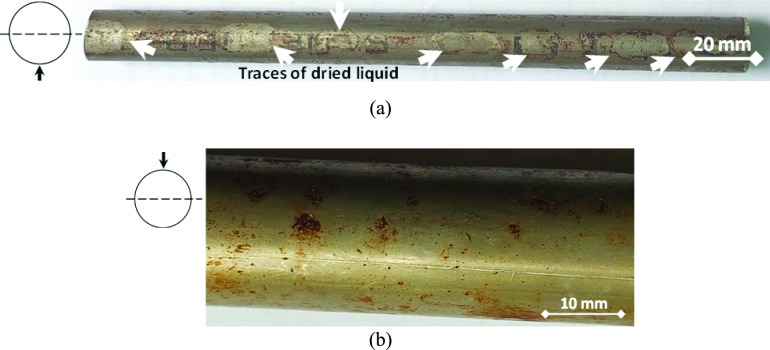
External surface of a leaking tube: (**a**) lower part of the tube external surface, where traces and indications of dried liquid are seen, and (**b**) upper part of the tube external surface, where no traces or indications of dried liquid are seen.

During the operation of the heat exchanger, the cooling water passes inside the tubes to cool the carbon dioxide containing gas, which passes around and in between the tubes. The visual examination images of the selected samples are shown in Fig. 4 to Fig. 6. Figure 4 shows the images of a retrieved leaking tube of the heat exchanger. Figure 4 (a) illustrates the lower portion of the external surface of the tube at the 6 o’clock position, and shows traces and indications of dried liquid. The upper portion of the external surface of the tube at 12 o’clock position shows neither traces, nor indications of dried liquid, as can be seen in Fig. 4 (b). This indicates that a liquid phase condensation took place at the external surface of the tubes, and the condensed liquid gathered down at the bottom position of the tube due to gravity. The gathered liquid wetted a width of 7 to 11 mm of the tube external surface along the tube length.

**Fig. 5:**
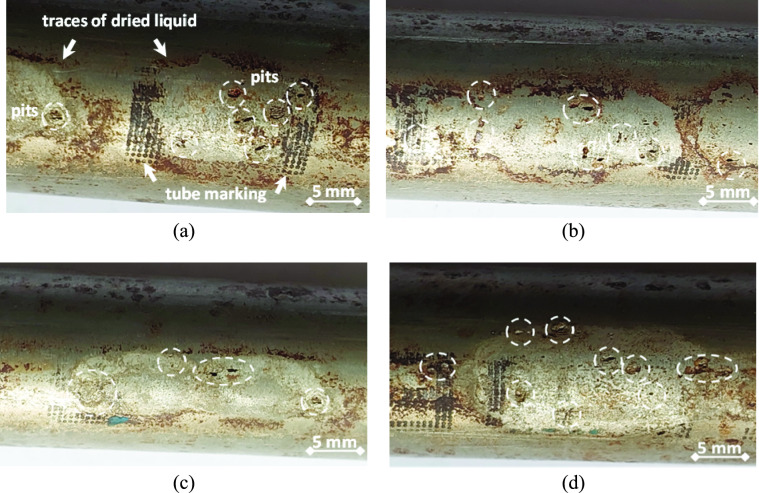
Pitting at the lower part (at about the 6 o’clock position) of the tube external surface: (**a**-**d**) close-up views showing pitting damages at several locations.

Figure 5 displays close-up views of the lower portion of the external surface, and indicates numerous pits (circled) at the lower portion of the tube. It can be observed that the pits exist exclusively within the trace areas of the dried liquid. This indicates that the condensed liquid played a major role in the pitting process of the stainless steel tubes.

**Fig. 6:**
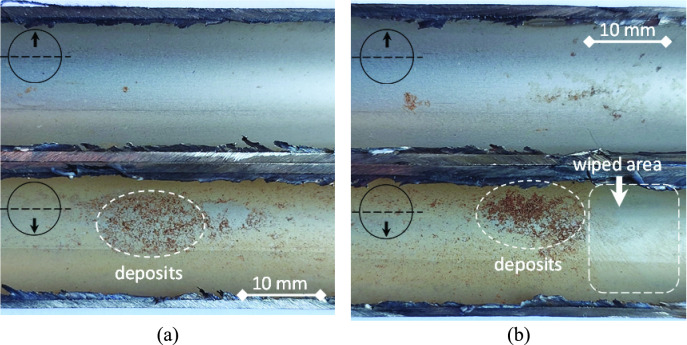
Images of the internal surface of the tube: (**a**) upper and lower zones of the tube internal surface, and (**b**) part of deposits was wiped away showing no pitting or corrosion underneath.

The retrieved leaking tube was cut axially into two halves at the 3 and 9 o’clock positions. The images of the internal surfaces of the two halves are shown in Fig. 6. It is clear that the lower portion of the tube internal surface shows a yellow color, and contains brownish deposits as can be seen in Fig. 6 (a). Part of the brownish deposits was wiped away using a dry cloth. The tube surface underneath the deposits was almost free from pitting or corrosion as shown in Fig. 6 (b). This indicates that the cooling water that flowed inside the tubes did not induce any negative effect or cause any corrosion damage. This suggests that the main pitting attack and the subsequent leaking occurred from the external surface of the tubes.

It was initially thought that the Microbiological Influenced Corrosion (MIC) might be a contributor to the reported leaking damage. The MIC refers to the microbial activities causing or inducing corrosion damage for metals and nonmetals^22^. MIC is one of the major damage mechanisms in oil and gas industry. It is responsible for about 20% of the total corrosion losses in that industry^23,24^.

It was believed that the circulating cooling water system may have scales and microorganisms, and via increasing scales, this can reduce the heat exchanger efficiency, and even lead to corrosion and perforation of the equipment^25,26^. However, this believe was completely denied by performing the visual examination of the leaking tubes, as it became clear that the damages were totally on the external surface. In addition, no internal corrosion was observed as shown in Fig. 5 and Fig. 6. Furthermore, it was ensured that the water used for cooling inside the tubes of the heat exchanger was condensate water. Therefore, there were no impurities, scales or bacteria that might exist and cause damage.

### Macroscopic examination

**Fig. 7:**
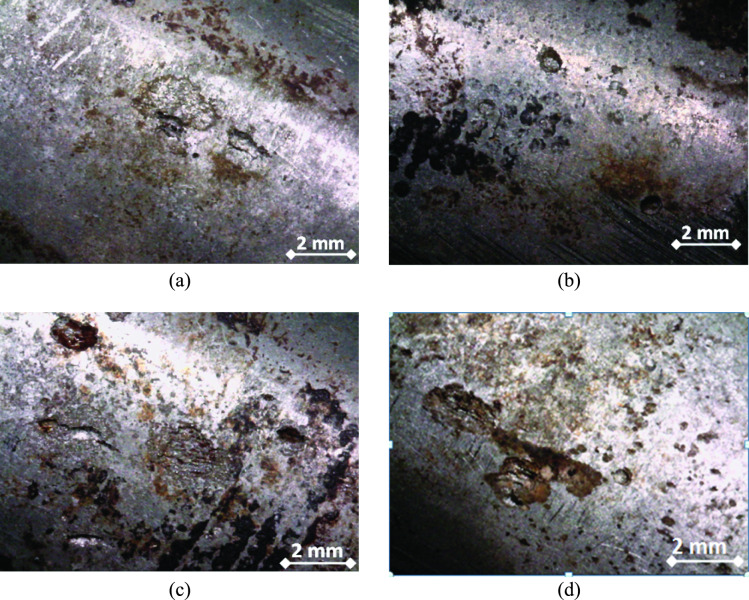
Macrographs of the pitting damage on the external surface of tubes: all pits of (**a**-**d**) are located within the dried liquid trace areas.

The second step of this study is the macroscopic examination. In this step, a stereomicroscope device was used to give magnified photos for the leaked sample. Modern stereomicroscopes can give magnification ranges from 10X up to 50X, which may be very useful in the macroscopic examinations. Figure 7 illustrates the macrographs of the pitting corrosion at the external surface of tubes. All pits exist within the traces of dried liquid as mentioned previously. It can also be seen that the pits have various and irregular shapes. Pit sizes are generally in the range of 1 to 2 mm. The localized pits might indicate a breakdown of the passive layer at certain locations. This might be due to certain ions in the liquid phase.

### Microscopic examination

Microscopic examination was done using the light optical microscope at larger magnifications than those of previous visual and macroscopic examinations. Magnifications in the range of 100X to 1000X are the most common. The optical microscopy provides additional important information. These include the uniformity and size of the grain structure, the size distribution and shape of intermetallic particles and inclusions, laps, folds, seams, porosity, presence of any external cracking and presence of oxygen diffusion layer on the surface.

**Fig. 8:**
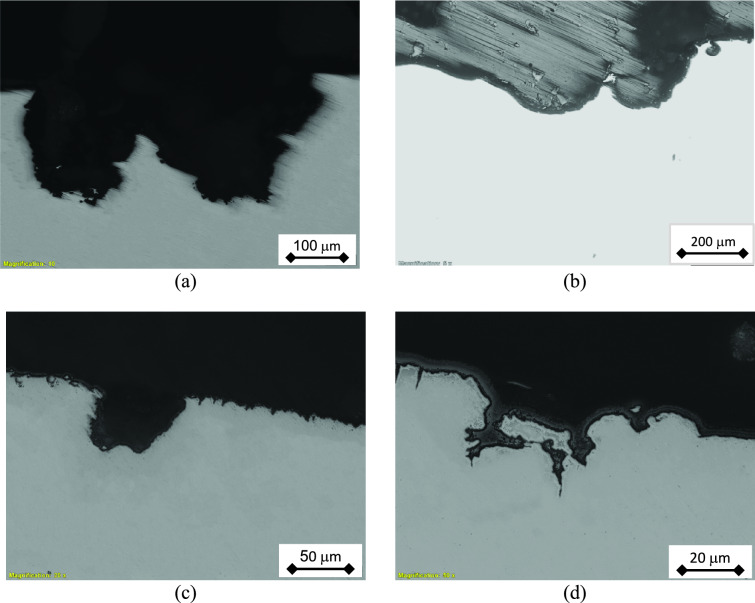
Optical micrographs of the pits at the cross section of the tube wall (un-etched). It is clear that pits with different profiles and depths are seen: (**a**-**c**) round pits, and (**d**) sharp pit.

The pitting at the external surface of the tubes is therefore investigated via the light optical microscope. Firstly, the tube was sectioned at several pit locations and the cross section was prepared by metallographic techniques. Figure 8 illustrates the micrographs of un-etched cross section of the tube at pit locations. It is clear that the pits exhibit various depths and several profiles, including round and sharp shapes.

Usually, when the fracture surface is microscopically examined, the damage tends to propagate within the grains as transgranular damage, or through the grain boundaries as intergranular damage^27^. The austenitic stainless steels can exhibit intergranular corrosion for many reasons, the most prominent ones are the differences in chemical composition and internal stresses of the metal^28,29^. This could occur after heating the surface of the austenitic stainless steel to a temperature range of 400° to 800°C during service, or upon welding in the heat affected zone (HAZ). This makes carbon to diffuse rapidly with chromium near the grain boundary, and form chromium carbide precipitates, leaving the areas near the grain boundaries depleted of chromium. This process is known as sensitization. The difference of chemical composition between the grains and grain boundaries leads to potential difference. Therefore, with the presence of appropriate corrosive environment, intergranular corrosion can occur^30,31^.

**Fig. 9:**
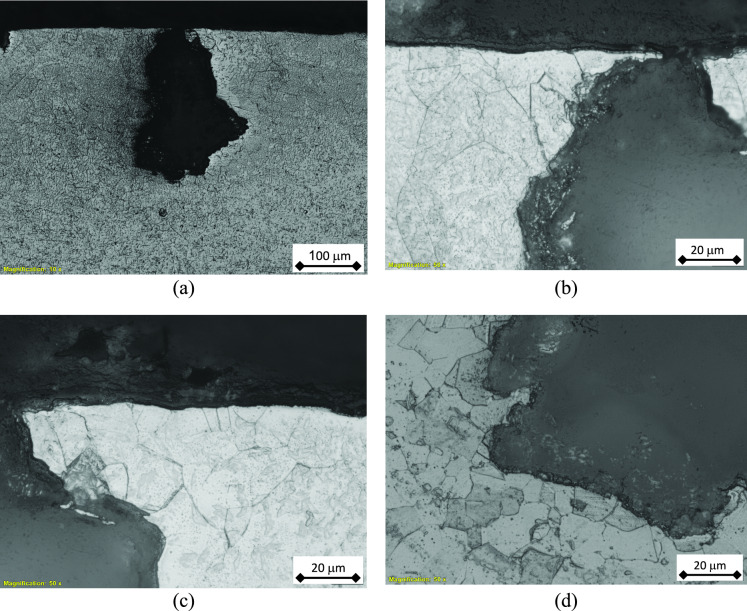
Bottle-like corrosion pit: (**a**) general view, and (**b**-**d**) close-up views showing corrosion propagation through grains.

The microscopic examination of the samples indicated that the failure did not propagate along the grain boundaries as illustrated in Fig. 9. It shows a typical pit profile with narrow opening at surface, large and lateral propagation under surface. It is a bottle-like pit profile. It is also clear that the corrosion propagated through dissolution of the grains, rather than the intergranular regions. Thus, there is no preferential path/direction of the corrosion attack. Furthermore, the leaked tubes were of 316L stainless steel, which can resist the sensitization phenomenon due to its lower carbon content^32^. The corrosion propagation via the intergranular path was not observed in Fig. 9.

**Fig. 10:**
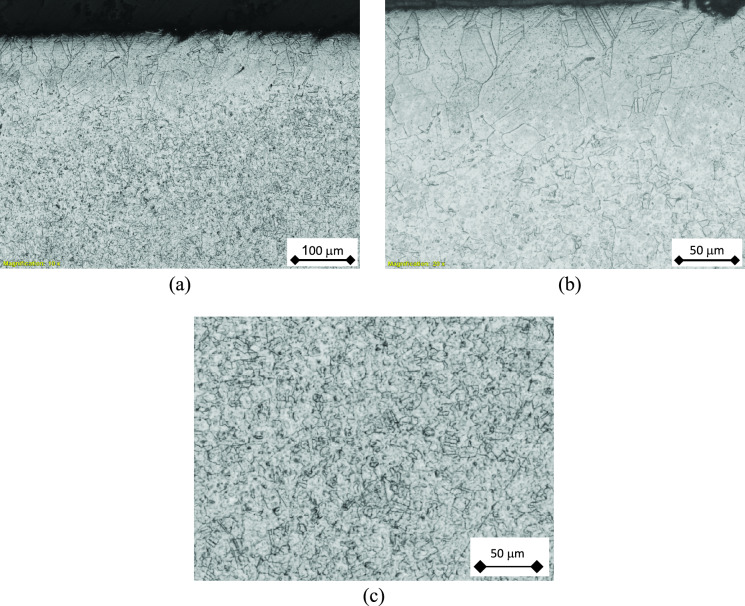
Optical micrographs of the cross-section grain structure of tubes: (**a**) dual grain size structure, (**b**) higher magnification of the dual grain size structure, (**c**) middle region showing fine second phase particles.

Figure 10 shows the dual grain size condition of the tube cross section. It is clear from Fig. 10 (a) that a very fine austenitic stainless-steel microstructure is dominant at the tube cross section. This is enveloped with a coarse grain layer at the external and internal tube surfaces, Fig. 10 (b). The coarse grain size area is likely formed during a subsequent solution annealing process. The fine-grained area shows numerous second phase particles of a round morphology inside the grains, as can be seen in Fig. 10 (c). This dual gain size indicates to an improper control of the heat treatment process during manufacturing the tubes. The large variation of grain size and the second phase particles might have an impact on the corrosion resistance of the tubes, as is discussed below.

A previous work confirmed the relation between annealing temperature and the pitting corrosion behavior of the 316L stainless steel^33^. It was reported that there were improvements of the pitting corrosion resistance of the 316L stainless steel, at an annealing temperature range from 800° or 900° to 1000°C, due to the homogenization of the microstructure, and the thicker passive film of the alloy^34^. Annealing temperature of 1050° up to 1200°C causes a drastic decline in the pitting resistance of the 316L stainless steel^35^. Therefore, the improper control of the temperature, time or the cooling rate of the annealing treatment of 316L stainless steel can affect its pitting corrosion resistance^35^. It is believed, accordingly, that the current tubes were improperly heat-treated during manufacture as indicated by the dual-grain size structure of Fig. 10. Therefore, the pitting corrosion resistance of the tubes might have been affected.

### Scanning electron microscopic examination (SEM)

In spite of the importance of optical microscopy in the current study, it focuses on a very narrow region because of the very shallow depth of field. Most of damages are complex, so that the powerful scanning electron microscopy with extreme depth of focus and high magnification is highly desirable. It can be operated by different modes: the most common being the secondary electron imaging, which provides a detailed high depth of focus that is easy to interpret. The other modes of SEM include the backscattered contrast, which is used to identify the regions of impurities responsible for the appearance and brightness of the image. The topographic backscattered mode enhances the surface topography of the sample, and clarifies the height differences of the fracture surface.

**Fig. 11:**
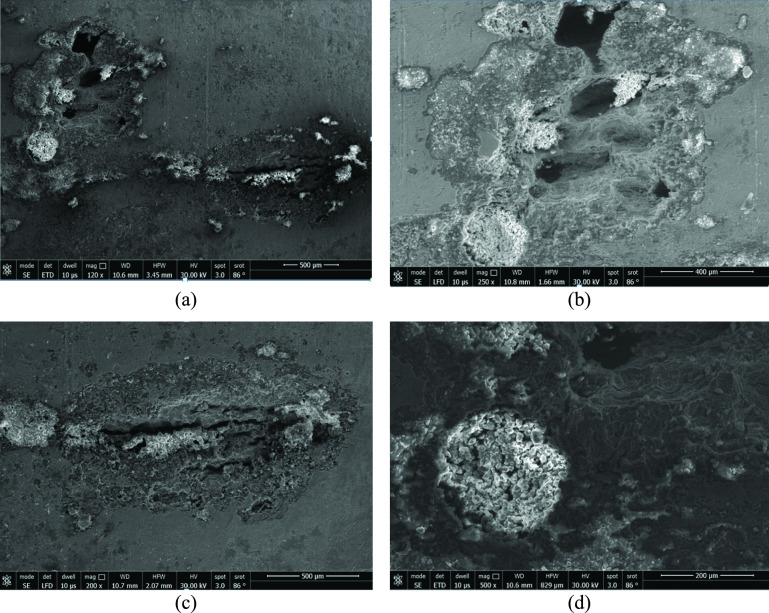
Secondary electron micographs of the external pitted surface of the tube: (**a**) area with sevral pitts, and (**b**, **c** and **d**) close-up views showing the various pit shapes and white contrast areas around pits.

Figure 11 shows the secondary electron micrographs at the external surface of the leaking tube. Figure 11 (a) shows general view of the pitted surface, while Fig. 11 (b to d) shows close-up views of various pit shapes and sizes. The pit sizes ranges from 1 to 2 mm, as can be estimated from the micrographs. White contrast areas are also seen within the pit locations. The white contrast areas are fine-grained equiaxed particles that are attached to the tube surface.

### Quantitative corrosion metrics

The dimensions of 15 large pits that were observed on the surface of the tubes are given in Table 3. These measurements were made using several imaging devices, such as the optical microscope, the stereomicroscope and the SEM. Part of the measurements was for the surface dimensions, while others are for the cross-sectional profiles of a cut surface. It is clear that the pits take various shapes and profiles, namely, irregular, elongated, bottle-like, sharp and round shapes. The sizes and depths of the pits show considerable variations as well. Some pits measure few millimeters and others are much smaller. These pits occurred in a period of about 70 days of service, during which leaking occurred. This indicates the severity of corrosion damage that affected the heat exchanger tubes.

**Table 3:** Quantitative pit dimensions.

Pit number	View, imaging device	pit dimensions, μm					Pit shape/profile
		length	width	depth	neck	diameter	
Pit 1	cross-section, microscope		583	255			round profile
Pit 2	cross-section, microscope		1000	194			round profile
Pit 3	cross-section, microscope		92	67			round profile
Pit 4	cross-section, microscope		69	36			sharp profile
Pit 5	cross-section, microscope		206	286	40		bottle-like profile
Pit 6	surface, stereomicroscope	2510	1640				irregular shape
Pit 7	surface, stereomicroscope	2680	570				elongated shape
Pit 8	surface, stereomicroscope	5620	2050				irregular shape
Pit 9	surface, stereomicroscope	1730	640				elongated shape
Pit 10	surface, stereomicroscope	1090	730				irregular shape
Pit 11	surface, stereomicroscope	1360	880				irregular shape
Pit 12	surface, stereomicroscope					730	round shape
Pit 13	surface, stereomicroscope					720	round shape
Pit 14	surface, SEM	1813	1250				irregular shape
Pit 15	surface, SEM	1482	821				elongated shape

### Energy dispersive spectroscopic examination (EDS)

The EDS microanalysis allows a quantitative elemental characterization of a given phase or within a microscopic zone of the surface. The presence of a specific element can be detected by its characteristic X-ray wavelengths, while the quantity of the element can be detected by the X-ray amplitude. The primary electron beam can be focused down to a spot of about one micron in diameter, allowing very small particles and phases to be analyzed for their chemical composition.

The EDS microanalysis in the current investigation was obtained for the external surface of the leaking tube. The EDS analyses of four locations are shown in Table 4. These are two locations at surface pits, one location at the white contrast area, and one location at sound tube area (*i.e.,* free from pits or attached particles). It is clear that high percentages of C, Cl, S, and F ions are analyzed in these locations. The analysis of the white contrast area of Fig. 11 (d) shows that it is mainly composed of calcium oxide particles, as can be seen in the EDS analysis of Table 4, where Ca was analyzed as 44% in this area.

**Table 4:** EDS analysis of the external surface of the leaking tube, wt. %.

Elements	Pit of Fig. 11 (b)	Pit of Fig. 11 (c)	Sound area of Fig. 11 (a)	White contrast area of Fig. 11 (d)
C K	13.45	13.51	3.94	9.26
O K	9.15	6.87	2.74	32.54
F K	1.14	1.30	-	-
Na K	1.32	2.13	0.74	0.54
Mg K	0.56	0.33	0.18	0.73
Si K	2.15	0.97	0.72	2.96
S K	1.01	0.62	0.66	1.46
Cl K	0.79	0.94	0.37	0.36
Ca K	8.53	2.00	0.79	44.08
Cr K	12.18	14.36	17.72	1.65
Fe K	42.93	49.56	61.45	4.93
Ni K	5.14	6.01	8.24	-
F + Cl	1.93	2.24	0.37	0.36

### Chemical analysis

The chemical composition of the tube is shown in Table 5. This analysis was obtained using an optical emission analyzer. The composition of the tube was compared with the standard composition of stainless steel 316L. The analysis of Ni, Mo and N are at their lowest permissible levels according to ASTM SA 213/ SA 213M Standard Specification^36^. This might keep the tube within the specification of the stainless steel 316L, but would not guarantee the anticipated corrosion and pitting resistance in harsh conditions. The current service conditions with high levels of halide ions and sulfur compounds would require higher grade alloy than the current 316L material.

**Table 5:** Chemical composition of the leaking tube.

Part	Element, wt. %									
	C	Si	Mn	P	S	Cr	Mo	Ni	Co	N
Tube	0.01	0.41	1.37	0.029	0.004	17.3	2.02	10.3	0.26	0.02
St. St 316L	0.03 max	0.75 max	2.00 Max	0.045 max	0.03 max	16 -18	2–3	10 -14	–-	0.10 max

## Discussion

### Mechanism of dew point corrosion

The current investigation deals with a leaking of two heat exchangers, after only 70 days of service in a sugar factory. Both units were put out of the service during the investigation of the root cause of their leakage. The heat exchanger tubes were made of 316L stainless steel. The units were used for cooling the CO_2_-gas resulting from the calcination of lime. The gas enters the exchanger at a temperature of 120–140°C, and exits at 55°C. Cooling water passes inside the tubes to cool the CO_2_-gas, which passes outside the tubes. The CO_2_-gas contacts only the outer surface of the tubes. The visual images of the leaking tube showed that there was no pitting in the internal surface of the leaking tube. The external surface of the leaking tube contained, alternatively, numerous pits of various sizes and shapes. The damage was clear on the external surface of tubes. The EDS analyses of the corrosion pit showed high levels of C, Cl, F and S ions.

In presence of high levels of the water vapor resulting from combustion, these elements tend to dissolve in the vapor phase upon cooling and form acidic flue vapors. These are H_2_SO_4_, HCl and HF, in addition to the carbonic acid (H_2_CO_3_.) Upon cooling, these acidic vapors condense as a liquid phase on the external surface of the heat exchanger tubes. The H_2_SO_4_ condenses at 138°C, the HCl at 54°C, the H_2_CO_3_ at 149°C^37^ and the HF at 111°C^38^. The multicomponent liquid phase caused corrosion and pitting damage to the tube surface in a very short period of service. This process is called “flue gas dew point corrosion”.

The term “dew point” refers to cooling the flue gas at constant pressure and constant vapor content to achieve the saturation state. Thus, any further cooling of the flue gas can result in formation a liquid phase, and at very low temperatures it can form a solid phase. Therefore, upon cooling or when the steel surface temperature becomes lower than the dew point of the acidic gas, the gas phase will condense forming acidic solutions on the steel surface. The accumulation of these aggressive solutions may lead to corrosion damage of the material surface^39^.

It is common that the researchers attribute the dew point corrosion exclusively to the H_2_SO_4_ condensation more than the other gases^40^. However, in the current study it was found that there were high levels of halides (Cl + F) in pit locations, as well. These levels are 1.93 and 2.24 Wt. % as can be seen in Table 4. On the other hand, the sulfur level does not show a trend between these locations. This indicates that the pitting damage is largely affected by the halide level, and to less extent by sulfur level.

### Role of halides

In the following paragraphs, the effect of HCl, HF and their synergistic effect will be addressed.

#### HCl corrosion

Many researchers studied the effect of HCl on the austenitic stainless steels, and particularly on the 316L grade. It was found that both of the 304 and 316 types are nonresistant to HCl at all concentrations and temperatures^41^. It is known that the 316L steel will crack in 5% HCl at temperature of 0°C. At high ambient temperatures, the corrosion rates are high. However, the presence of nickel and molybdenum may show some resistance in dilute HCl solutions. Even though, pitting, local attack and stress corrosion cracking may occur in these conditions. The corrosion products, especially FeCl_3_, can cause cracking. The presence of chlorides can result in penetrating and destroying the passivity (oxide film) that is responsible for the corrosion resistance of the stainless steels. The 316L alloys show little resistance to HCl compared to other materials, such as the super austenitic stainless steels, and the higher nickel alloys^39^.

#### HF corrosion

There are several factors affecting the corrosion rates of steels in the HF solutions. These are the flow velocity, the concentration and the temperature of the HF solution. It is reported that the hydrofluoric acid vapor will condense on the surfaces with concentration of 38% HF, when the surface temperature is at a dew point of 111°C. The concentration of the condensed liquid phase will be water enriched or fully HF enriched. If the temperature is below the dew point and the concentration of the vapor is lower than 38% HF, the condensed phase will be water enriched. While when the concentration of the vapor is higher than 38%, the condensed phase will be HF enriched. Austenitic stainless steels show resistance only at dilute HF. Type 304 stainless steel show very poor resistance to concentrations higher than 1% HF, and with 5% HF the 304 alloys show higher corrosion rates than carbon steels at the same conditions. Type 316 stainless steel show good resistance only at 23°C or lower temperatures, at 10% HF concentration or less. It is found that the 316L alloys show higher corrosion rates when exposed to higher concentrations and higher temperatures of HF^38^.

#### Synergistic effect of HCl and HF

The effect of HCl and HF on the corrosion of austenitic stainless steels particularly type 316L is addressed in these paragraphs. Fluorides and chlorides are known as strong corrosive species of metals when existing in the same media. Few works studied the relation between the existences of halide ions (Cl + F) in the same environment, and covered the corrosion of the austenitic stainless steels and in particular the 316L alloys. It is known that the industries that involve combustion processes usually have emissions of fluorides and chlorides in the form of HF and HCl flues gases, besides the sulfur oxides in the form of H_2_SO_4_. This creates strong aggressive media products on the material. It is known that the 316L stainless steel shows some resistance to the fluorides at certain conditions more than the chlorides, unless both ions are found in the same media^42^.

Most of stainless steels have poor resistance of the hydrohalic acid (which contains both chlorides and fluorides), since they are unable to preserve their passive chromium oxide film on their entire surface. The presence of halides in the same media leads to active local corrosion due to their physicochemical properties. It is reported that the fluoride ions are usually surrounded by tight hydration shells, and tend to induce general corrosion. While the chloride ions possess loose hydration shell, and induce pitting corrosion. In more details, the chloride has a noticeable effect on the activation domain, whereas the HF affects the passive domain. Therefore, the fluorides attack results in the formation of semi soluble CrF_3_ product, while chlorides dissolve the chromium oxides of the passive film to soluble Cr^3+^. This can be described as a synergistic effect that HF induces the corrosion of the passive film affected by the existence of chlorides^42^.

This means that chlorides facilitate the HF penetration into the passive film. It is reported that several metals, including stainless steels, carbon steel and magnesium alloys, tend to form passive films from the corrosion products during the corrosion process to prevent further corrosion. However, fluorides restrict the passive film growth, and then affect the structure of the film, leaving the bilayer film structure into a monolayer structure. This reduces the resistance of the passive film and degrades the protection of the alloy in the environment. This occurred with high concentrations of chlorides and above 1% of HF. Therefore, the existence of halide ions (both of chlorides and fluorides condensing from the acidic flue gases) in the same environment accelerates the corrosion damage of austenitic stainless steels, similar to the observations of the current study. These explanations are useful to understand the tube leaking in the presence of high levels of chlorides and fluorides^43,44^. The EDS analysis of halide ions in the pit locations would, therefore, indicate their synergistic corrosion effect and explain their major role in the pitting of the 316L tube. The role of F ions in the pitting process is now clarified, as well.

### Role of carbonic and sulfuric acids

#### CO_2_ corrosion

As mentioned previously, the presence of halide ions plays the major role in the pitting process of the current 316L stainless steel tubes. Besides HCl and HF, the flue gas contained high levels of CO_2_ and H_2_SO_4_. Both CO_2_ and H_2_SO_4_ have lower impact on the pitting process compared to the HCl and HF. However, their effect cannot be neglected. The dew point of CO_2_ in form of carbonic acid is 149°C, whereas the flue gas temperature upon entering the heat exchanger was from 120° to 140°C. Therefore, it definitely underwent condensation while passing inside the heat exchanger. The existence of CO_2_ combined with the halides at high temperature can affect the pitting rate. Many researches agreed that when the 316L stainless steel is exposed to a moisture enriched with CO_2_ gas with/without Cl^-^ ions and H_2_S, this affects the pitting resistance of 316L austenitic stainless steel^45,46^. This multicomponent phase is similar to that encountered at the surface of the tubes in the current study.

Ezuber studied the effect of temperature on the pitting corrosion behavior of the 316L stainless steel when exposed to chloride-CO_2_ environment. It was found that the pitting potential of the alloy decreased with increasing the chloride concentration and increasing temperature. It was also found that the CO_2_ has no significant effect on pitting at low temperatures of about 25°C. However, upon raising temperature to 80°C, the CO_2_ played an important role in increasing the stability of chloride ions on the surface of the alloy, and gives negative shifts of the pitting potential in the direction of accelerating the pitting rate. Therefore, the CO_2_ has a significant effect on inducing the pitting rate of 316L stainless steel, at high temperature in presence of high concentrations of chlorides^47^. This is almost identical to the conditions of the current investigation where high levels of CO_2_ exist. The role of H_2_SO_4_ in the pitting of the 316L tubes is discussed in the following paragraphs.

#### H_2_SO_4_ corrosion

The effect of H_2_SO_4_ in inducing the pitting process cannot be neglected, even it is less pronounced compared with the other acidic vapors in the flue gas. The amount of H_2_SO_4_ in the flue gas is dependent on the sulfur content of the fuel. During the combustion process, the sulfur is oxidized into SO_2_. Then, small amount of SO_2_ transforms to SO_3_, depending on the furnace temperature and the residence time. Upon decreasing the gas temperature below 500°C, the SO_3_ starts to react with water vapor and form H_2_SO_4_. At about 200°C, the SO_3_ content of the gas is completely transformed to H_2_SO_4_. Therefore, when the steel surface temperature is below the dew point of H_2_SO_4_ that is approximately 138°C, the vaporous acidic gas of H_2_SO_4_ begins to condense on the steel surface. The amount of deposited sulfuric acid on the steel surface is mainly dependent on the temperature, the amount of water vapor and the concentration of SO_3_^48,49^. The current conditions are suitable for the condensation of H_2_SO_4_, since the gas goes from 140 to 55°C inside the heat exchanger.

At temperatures above the dew point of the acidic gas, the sulfuric acid can deposit in concentrated form or stored in form of sulfate deposit. On cooling, sulfuric acid absorbs moisture from air and dilutes its concentration down to the ambient temperature. It is reported that the corrosion resistance of austenitic stainless steels in sulfuric acid environment is somewhat complex. It was found that both of the 304 and the 316 stainless steels can maintain their passivity in stable state in the very low and the very high concentrations of sulfuric acid. Therefore, the 316 stainless steel can be used for 93% acid concentration at ambient temperature of ~ 40°C. While at 98.5% acid concentration, the ambient temperature should above 70°C. At 99% H_2_SO_4_ concentration, the corrosivity decreases rapidly allowing the use of stainless steels above 100°C^50^. Even though, synergistic effect of the four acidic components might play the overall role of damage, it seemed that the roles of HCl and HF were more pronounced. These findings support the results of the current investigation as the corrosion damage of the tubes is mainly due to the condensation of both HCl and HF, and to less extent to H_2_CO_3_ and H_2_SO_4_.

### Physical effects

The CaO particles of Fig. 11 and Table 4 played a role in the observed corrosion of the tubes. These particles seem to be physically attached to the pitting area of the tubes via wetting with the liquid phase. In this respect, the particles likely have played an indirect role in maintaining the tube surface wet with larger amount of the corrosive liquid phase, and for longer time as well. Apart from this physical role, there is no visual evidence of any chemical role in the pitting process. These fine particles were transported from the lime calcination furnace to the heat exchanger via the CO_2_ gas. Thus, the particles were of external source, and played an indirect and important physical role in the pitting attack.

### Remedies

After discussing the main conditions of pitting of the 316L stainless steel tubes in the current investigation, it can be seen that the dew point corrosion is a significant problem facing most of the heat exchangers. This phenomenon occurs when the heat exchanger cools flue gases enriched with harmful acidic vapors, which usually result from the combustion of fuel containing impurities. Therefore, many works studied the dew point corrosion phenomenon, and tried to reduce or eliminate it via different solutions. Thermal insulation or coating was the first way used to reduce the effect of the condensing acidic gases on the heat exchanger material. The advantage of this method is to reduce the steel surface temperature, then reducing the thermal damage, and allows using affordable materials. However, the temperature gradient between the outside and inside of the insulation decreases the efficiency of the heat exchanger. Besides, the condensed acidic solutions, *e.g.,* HCl and H_2_SO_4_, can diffuse through the coating layer over time, and can eventually cause corrosion. This makes coating and insulation ineffective methods to solve the dew point corrosion problem^39,51^. Other remedies such as material selection are discussed in the next sections.

### Modeling insights

Predicting the acidic flue gas dew point is the most common way to reduce the effect of dew point corrosion. There are three basic methods for predicting the acidic flue gas dew point: the direct measurement methods, the empirical methods and the semi-empirical methods. The direct measurement methods are based on a conductivity sensor, whose temperature can be modified by a flow of cold air. The sensor can detect the signal after accumulation of a considerable amount of condensed acid. Thus, this makes the signal lags more or less, so that different lagging leads to different detection results^52^. The empirical and semi-empirical methods are different. The empirical models are simple, have a single formula, and are established by data fitting between the input parameters and the results. They do not reflect any physical principle. On the other hand, the semi-empirical models are difficult, complex, and dependent on the thermodynamic laws and the equilibrium theory of gas–liquid mixtures. So that, each formula has a physical meaning. The formulas of the empirical models have different input parameters.

As mentioned previously, most researches attribute the dew point corrosion to the condensation of H_2_SO_4_^38,40^. Therefore, the major efforts focused on predicting the dew point of the H_2_SO_4_, while predicting the dew points of the other acidic gases was substantially ignored. As a consequence of this, most of the empirical models were developed to predict the dew point of the H_2_SO_4_; and hence most formulas were dependent on the input parameters, which include the sulfur content in fuel, the SO_3_ concentration in the flue gas, the H_2_SO_4_ concentration in the flue gas, the ash content in coal, and the H_2_O vapor content in the flue gas^49^.

The current investigation uses the H_2_O vapor content in the flue gas as an input parameter for predicting the different acidic flue gas equations. The equations, and the relation between the temperature of the flue gas and the partial pressure of the condensed acidic phase are shown in Appendix A.

It is visibly clear from Figure A1 (in Appendix A) that the partial pressure of SO_3_, that is needed for condensation of the H_2_SO_4_, is proportional to lowering the temperature of the flue gas. In other words, upon cooling, the values of the partial pressure of sulfur trioxide, which dissolves in water to form sulfuric acid decreases. This results in condensation of dilute sulfuric acid on the tube surface as mentioned previously. Thus, the gas partial pressure is directly proportional to the acid concentration. This means that high values of partial pressure lead to high concentrations of the condensed acids^53^.

Apart from the sulfuric acid condensation, the HCl and HF diagrams of Figure A1 give obvious results, and insights on the effect of the flue gas temperature on the partial pressure of the acidic gases required for condensation on the tube surfaces. The flue gas temperatures investigated are the same of the heat exchanger operating conditions. Figure A1 shows that the highest value of the partial pressure of HCl, that is 5.03 mm Hg at 40°C (*i.e.,* 0.702 at the log scale axis), is below its dew point. The diagram shows clearly that with lowering the temperature of the flue gas, the partial pressure for condensing HCl increases. The dew point of HCl is 54°C, below which higher concentrations of the HCl solution are obtained on the tube surfaces. Similarly, Figure A1 shows that lowering the flue gas temperature below the dew point of the HF vapor (*i.e.,* 111°C), results in an increase in the concentrations of condensed HF. As expected, the highest partial pressure for condensation of the HF occurs at the low flue gas exit temperature (*i.e.,* 50- 40°C) from the heat exchanger. This behavior is opposite of the H_2_SO_4_. Therefore, it becomes clear there is a synergistic effect of both HCl and HF, via condensation in high concentrations on the tube surfaces of the heat exchanger upon cooling below their dew points. This leads to rapid and severe corrosion of the cooling tubes, then to leaking incidents.

Additional valuable insights can be acquired from Figure A1, the first of these is the condensation behavior of the HCl compared to the H_2_SO_4_ and the HF solutions. It is clear that the HCl readily condenses even at very low partial pressures of the hydrogen chloride gas. In other words, the HCl acid is easier to condense than the H_2_SO_4_ and the HF acids. Thus, even at infinitely small levels of the HCl gas, condensation occurs. This makes the HCl the amplest component in the condensed liquid. Consequently, the observed damage is highly attributed to the HCl, due to its abundance as a condensed liquid over a large temperature range, and to its aggressiveness to the stainless steel passive layer.

The HF shows different behavior of condensation. Larger partial pressures are required for condensation, especially at lower temperatures. The existence of HF in the dried deposits on the tube surface in Table 4 indicates that the partial pressure of condensation was satisfied at certain temperature inside the heat exchanger. This likely occurred at 140–120°C, where the partial pressure of HF condensation that might be attained was reasonable.

The condensation of H_2_SO_4_ still shows different trend from those of HCl and HF. The H_2_SO_4_ condenses at a narrow range of partial pressures, *i.e.,* a relatively high level of partial pressures. This means that the lower concentrations of the H_2_SO_4_ gas than those indicated in the diagram, will not condense in a liquid form, and will remain in a gaseous form in the flue gas. Thus, their influence in the corrosion of the heat exchanger metallic tubing will be limited.

**Fig. 12:**
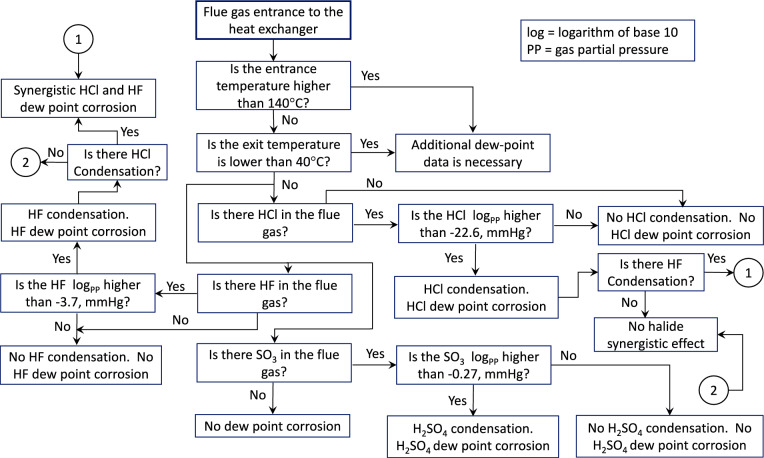
Flowchart illustrating the infleunce of temperature and gas composition on the likelihood of HCl, HF and H_2_SO_4_ dew point corrosion. The chart is constructed for heat exchanging applications in the temperature range of 140 to 40°C.

The flowchart of Fig. 12 was constructed based on the discussion of this section and the information of Appendix A. The chart aims at simplifying the influence of temperature and gas composition on the likelihood of HCl, HF and H_2_SO_4_ condensation. It also provides a decision-making tool, for the academic and industrial partitioners studying the flue gas dew point corrosion damages.

### Material selection implications

The last point of discussion is the chemical composition of the 316L tube. The chemical analysis of the leaked tube showed a chemical composition in conformity to the standard specification of the 316L stainless steel, *i.e.,* ASTM SA 213/ SA 213M^36^. However, the Ni, Mo and N levels were at their lowest permissible levels. It is known that the chemical composition of the stainless steels plays a major role, and affects their ability to resist pitting. One of the metallurgical parameters that are used for this assessment is the Pitting Resistance Equivalent Number (PREN), which is calculated based on the alloy chemical composition as follows:

1 $${\text{PREN }} = \, \% {\text{Cr }} + { 3}.{3}\left( {\% {\text{Mo}}} \right) \, + {16}\% {\text{N}}$$

The PREN is a calculated value that estimates the pitting corrosion resistance of stainless steels and other alloys. It depends on the alloy chemical composition, especially: Cr, Mo, and N. It was developed initially for the duplex stainless steels. The research and industrial societies use this parameter for assessment of the stainless steel pitting resistance. So that, higher values of the PREN indicate higher pitting resistance of the alloy against the chloride ions. It was also proposed that alloys with PREN of less than 40 are not suitable for aggressive environments. The chemical composition of the current tubes, with Ni, Mo and N at their lowest levels, gives a PREN value of 24.3, which is a very low value that would not guarantee pitting resistance in halide rich environments.

According to most industrial codes (*e.g.,* NACE, ASME, API, and ISO), for austenitic stainless steels grades 304 and 316, the widely accepted safe limit of the environment is ≤ 50 ppm chlorides. For critical applications of hydrotesting and high temperatures, stricter limits of < 30 ppm apply. Thus, the 316L stainless steel alloy would not be immune in the heat exchanger service conditions, and pitting would occur, especially when it serves in aggressive environment^54^. Recent reports of rapid dew point corrosion of stainless steel 316 tube bundles showed deterioration after 10 days of service of a flue gas reheater in a natural gas purification plant^55^. Thus, it is worth considering alternative alloys in this discussion, for better performance in the current service conditions.

Selecting or developing an alternative alloy for the multicomponent acidic environment might be a challenging task for the metallurgists and material developers, due to the multifaceted effects of the corrosion problem encountered in this case. However, providing novel alloys for this service is ultimately and technically necessary task. Apart from developing new alloys, there are some candidate materials that might perform better than the conventional 316 stainless steels.

For better pitting resistance in acidic mixtures, high performance alloys can be used. The candidate materials are (i) super-austenitic stainless steels, (ii) duplex stainless steels and (iii) nickel base alloys. The super austenitic grades such as 254 SMO, with PREN of 42–46, offer better performance than conventional stainless steels. However, it may still suffer in aggressive halide environments rich in HF, since the HF attacks the Mo/Cr passive layer. The duplex stainless steel grades provide a good balance of strength and corrosion resistance, but not suitable for extreme levels of halides. The super duplex stainless steel 2507, with PREN of ~ 42, might perform better than the 316L. However, the duplex grades may suffer pitting/crevice corrosion in highly hot concentrated chlorides media^56^. The super austenitic and duplex stainless steel are cost-effective options for moderate conditions.

The nickel base alloys show superior resistance to halides, particularly in extreme conditions. Several alloys can be used, *e.g.,* Hastelloy C-276, Hastelloy C-22, Inconel 686, Alloy 59, and Hastelloy C-2000. The PRENs of these alloys range from 70 to 80, indicating resistance to extreme pitting conditions. These alloys offer outstanding resistance to halide attack and concentrated acidic solutions of HCl and HF^57–61^. However, the prime limitation of using these alloys is their high cost compared to the duplex and the austenitic stainless steel grades.

## Conclusions

It became clear from the results of this investigation that the main damage mechanism of tube leaking of the heat exchangers was the flue gas dew point corrosion. The condensations of hydrochloric, hydrofluoric and sulfuric acids on the cooling tubes of the heat exchangers caused pitting damage, besides the role of the carbon dioxide in increasing the pitting rate. The existence of both hydrochloric and hydrofluoric acids in the same environment weakened the alloy passivity and made it unable to resist the pitting damage. This is known as the synergistic effect of the halides. The results of the investigation show that lowering the temperature of the flue gas increases the concentrations of the condensed acidic gases of HCl and HF on the tube surface. This leads to rapid and severe corrosion damage. This explains the rapid degradation and leaking of the heat exchanger tubes after a short service life of only 70 days.

The tube composition is not suitable for the dew point corrosion conditions, where hydrochloric and hydrofluoric acids are the major aggressive species. Therefore, the root cause of corrosion of the 316L stainless steel tubes and the subsequent leaking in the heat exchangers was the improper material selection. Suitable candidate alloys for this service were proposed and justified.

## Supplementary Information

Supplementary Information.

## Data Availability

All Data generated or analyzed during this study are included in this published article.
