# Supplementary material for: Leaking of 316L stainless steel heat exchanger tubes in carbon dioxide process
Source: Sci Rep. 2025 Oct 7;15:35027. doi: 10.1038/s41598-025-21376-w (PMC12504518; doi:10.1038/s41598-025-21376-w)
Supplement: Supplementary file 1 — Supplementary Information. [file 41598_2025_21376_MOESM1_ESM.docx]

**Appendix A**

**Predicting the Flue Gas Dew Points**

In the current study, the H_2_O vapor content in the flue gas is used as an input parameter for predicting the different acidic flue gas equations, as shown in the literature [^[[1]](#endnote-1)^]:

| **Dew point of water vapor** $T_{g}=\frac{5038.13}{20.1424-\ln(P_{H_{2}O})}$ **Equation A1** |
| --- |

| **Dew point of sulfuric acid (H_2_SO_4_):**  $\frac{1000}{T}=2.276+0.02943\ln(P_{H_{2}O})-0.0858\ln\left( P_{SO_{3}} \right)+0.0062\ln(P_{H_{2}O})\ln(P_{SO_{3}})$ **Equation A2** |
| --- |

| **Dew point of hydrochloric acid (HCl):**  $\frac{1000}{T}=3.7368-0.1591\ln\left( P_{H_{2}O} \right)-0.0326\ln\left( P_{HCl} \right)+0.00269\ln(P_{H_{2}O})\ln(P_{HCl})$ **Equation A3** |
| --- |

| **Dew point of hydrofluoric acid (HF):**  $\frac{1000}{T}=3.8503-0.1728\ln\left( P_{H_{2}O} \right)-0.02398\ln\left( P_{HF} \right)+0.001135\ln(P_{H_{2}O})\ln(P_{HF})$ **Equation A4** |
| --- |

Where:

$T_{g}$ = gas temperature in (^◦^K).

$T$ = acid dew point in (^◦^K).

$P_{H_{2}O}$ = partial pressure of water, mm Hg

$P_{SO_{3}}$ = partial pressure of SO_3_, mm Hg.

$P_{HCl}$ = partial pressure of HCl, mm Hg.

$P_{HF}$ = partial pressure of HF, mm Hg.

Based on Equations A1 to A4, the relations between the temperature of the flue gas and the partial pressure of the acidic components are constructed, as shown Figure A1. The calculations cover the temperature range from 140°C, which is the entrance temperature, to 40°C, which is the exit temperature of the flue gas from the heat exchanger. The partial pressure of the water vapor is calculated as per Equation 1 for different gas temperatures. Then, each value of the partial pressure of the water vapor is used to predict the partial pressure of the acidic gases, namely H_2_SO_4_, HCl, and HF using Equations A2, A3 and A4. In doing these estimations, it was found that upon cooling, the partial pressure of the water vapor, that is necessary for condensation, decreased leaving a condensed liquid phase on the tube surfaces.

|  |
| --- |
| Figure A1: Relation between the temperature of the flue gas condensation and the partial pressure of the acidic gases. The curves are constructed based on Equations A1 to A4 for the SO_3_, HCl and HF gases. |

**References**

1. Yen-Hsiung Kiang, “Predicting Dew Points of Acid Gases”, Environment Energy Notes, Vol. 8, 2017. [↑](#endnote-ref-1)
